# Lower serum triglyceride level is a risk factor for in-hospital and late major adverse events in patients with ST-segment elevation myocardial infarction treated with primary percutaneous coronary intervention- a cohort study

**DOI:** 10.1186/1471-2261-14-143

**Published:** 2014-10-10

**Authors:** Yu-Tsung Cheng, Tsun-Jui Liu, Hui-Chin Lai, Wen-Lieng Lee, Hung-Yun Ho, Chieh-Shou Su, Chia-Ning Liu, Kuo-Yang Wang

**Affiliations:** Cardiovascular Center and Department of Anesthesiology, Taichung Veterans General Hospital, Taichung, Taiwan; Departments of Medicine and Surgery, Yang-Ming University School of Medicine, Taipei, Taiwan; Taipei First Girls High School, Taipei, Taiwan; Chung-Shan Medical University School of Medicine, Taichung, Taiwan

**Keywords:** Triglyceride, Myocardial infarction, Coronary, Revascularization, Restenosis, Outcome

## Abstract

**Background:**

Whether serum triglyceride level correlates with clinical outcomes of patients with ST segment elevation myocardial infarction (STEMI) treated by primary percutaneous coronary intervention (pPCI) remains unclear.

**Methods:**

From June 2008 to February 2012, all patients with STEMI who were treated with pPCI in this tertiary referral hospital and then had fasting lipid profiles measured within 24 hours were included and dichotomized into lower- (≦150 mg/dl) and higher-triglyceridemic (>150 mg/dl) groups. Baseline characteristics, in-hospital outcomes, and late major adverse cardiovascular events (MACE) were compared in-between. Independent predictors for in-hospital death and late adverse events were identified by multivariate logistic and Cox regression analyses.

**Results:**

A total of 247 patients were enrolled, including 163 lower-triglyceridemic and 84 higher-triglyceridemic subjects. The angiographic characteristics, pPCI results and in-hospital outcomes were similar between the two groups. However, multivariate logistic analysis identified triglyceride level as a negative predictor for in-hospital death (OR 0.963, 95% CI 0.931-0.995, p = 0.023). At follow-up for a mean period of 1.23 to 1.40 years, compared with the high-triglyceridemic group, low-triglyceridemic patients had fewer cumulative incidences of target vessel revascularization (TVR) (21.7% vs. 9.5%, p = 0.011) and overall MACE (26.1% vs. 11.9%, p = 0.0137). Cox regression analysis confirmed serum triglyceride as a negative predictor for TVR and overall MACE.

**Conclusions:**

Serum triglyceride level inversely correlates with in-hospital death and late outcomes in patients with STEMI treated with pPCI. Thus, when managing such patients, a high serum triglyceride level can be regarded as a benign factor but not a target for aggressive therapy.

## Background

Atherosclerotic cardiovascular disease remains the leading cause of disability and death in the Western world [1]. When coronary arteries are involved, ST-segment elevation myocardial infarction (STEMI) manifests an extreme presentation that carries ominous short- and long-term prognosis in terms of fatal arrhythmia, acute pumping failure, myocardial rupture and chronic left ventricular dysfunction [2]. Timely primary percutaneous coronary revascularization (pPCI) is well established as the most effective therapy for restoring vessel patency, salvaging jeopardized myocardium and preserving cardiac performance [2, 3]. However, a substantial proportion of patients are still at risk of early electrical/mechanical complications and late major adverse cardiac events (MACE) from myocardial stunning, unfavorable left ventricular remodeling, culprit lesion restenosis and de novo coronary stenosis [4], raising identification of high-risk patient subsets as an essential issue in the goal to improve the overall outcome of such a disease.

The chief prognostic determiners for patients with STEMI are the sizes of the infarcted and residual viable myocardium. When the culprit vessel is successfully opened by pPCI and the ischemic territory is thereby limited, a number of other factors may play a predictive role in determining the prognosis of these patients. Among such factors low-density lipoprotein (LDL) is closely related with early and late cardiovascular events [5] in patients with acute coronary syndrome; hence the use of aggressive statin therapy for these patients is justified to benefit cardiovascular outcome [6]. In contrast, whether serum triglyceride (TG) correlates with the outcome of STEMI and how it should be managed remains unclear. Some investigations showed that hypertriglyceridemia independently predicted development of coronary artery disease (CAD) [7, 8] and myocardial infarction [9] as hypercholesterolemia did [10], whereas others reported low TG levels were associated with poorer prognosis in some cardiac and non-cardiac disease states [11–13]. Given the controversial yet potentially crucial impact of hypertriglyceridemia on the outcome of patients with STEMI treated by pPCI, this study was designed to elucidate the differences in short- and long-term cardiovascular outcomes between patients with high- and low-serum TG levels. The results of this study could clarify the role of serum TG in determining the prognosis of patients suffering from STEMI, and may help guide proper management of such patients to improve their overall outcome.

## Methods

### Patients

From June 2008 to February 2012, of the 301 patients admitted to this tertiary referral center with a diagnosis of STEMI for primary PCI (pPCI), 247 had a lipid profile checked within 24 hours of admission so were enrolled and categorized into either the lower (≦150 mg/dl, N = 163, or 66%) or the higher (>150 mg/dl, N = 84, or 34%) triglyceride group according to the baseline level of serum triglycerides. Baseline characteristics, angiographic findings, and clinical outcomes were obtained from electronic or written medical records as well as the cardiac catheterization laboratory database and were compared between groups. The study protocol was approved by the human research committee of Taichung Veterans General Hospital (approval No. CE12289), and requirement of informed consent was waived by the institutional review board.

### Coronary angiography and percutaneous coronary intervention

The diagnosis of STEMI was made based on conventionally standardized criteria [2]. Coronary angiography was performed from either a radial or femoral approach at the discretion of the interventional cardiologists. Grade of blood flow was determined by TIMI classification system [14]. Angiographic stenosis was defined as a luminal diameter reduction of ≥50% by quantitative coronary angiography, and critical stenosis was defined as ≥70% narrowing of the coronary artery luminal diameter. Complete coronary occlusion was defined as absence of antegrade flow of contrast media beyond a specific vascular segment. The infarct-related lesion was determined by the relevant ECG leads showing ST-segment elevation (or ST-segment depression along with a tall R wave in lead V1 for posterior infarction) and by the typical vascular morphological features of thrombus-laden or hazy filling defects along with compromise of distal flow. Once angiographic occlusion or critical stenosis of any of the coronary arteries was demonstrated, PCI with or without stenting was performed for the infarct-related artery but not for any other bystander vessel unless hemodynamic instability persisted despite opening of the culprit lesion. The success of pPCI was defined as achievement of the TIMI flow of the infarct related artery to grade II or III. Patients were then taken to our intensive coronary care unit for post-procedural care. Pharmacological treatment, including dual-antiplatelet medication, was in accordance with current guidelines [2]. In-hospital complications, including cardiogenic shock requiring intra-aortic balloon pumping support, new-onset atrial fibrillation, ventricular arrhythmia, and death served as in-hospital outcomes.

### Follow-up

Discharged patients were followed up by medical records of clinic visits or telephone contacts for MACE, including data regarding non-fatal myocardial infarction, target vessel revascularization (TVR), need of coronary artery bypass grafting, cardiac death and all-cause mortality. Use of lipid-lowering agents (chiefly statins and fibrates) and non–lipid-lowering drugs (aspirin, clopidogrel, cilostazol, β-blockers, angiotensin-converting enzyme inhibitors, angiotensin receptor antagonists, aldactone receptor blockers) was compared between groups.

### Statistical analysis

Continuous variables were expressed as mean ± standard deviation (SD). Normally distributed continuous data were compared between groups by unpaired student’s T-test, while non-parametric continuous data were compared by Mann–Whitney U test. Categorical variables were compared by χ^2^ analysis with Fisher’s exact correction. Univariate followed by multivariate logistic regression analyses were used to identify independent correlates of in-hospital mortality in all patients. Rationale variables selected for regression analysis included the following: demographic characteristics, co-morbid illnesses, echocardiographic indicators, and angiographic features. Variables were tested in a backward conditioned multivariate logistic regression model if their univariate *P* values were <0.20. Statistical significance was defined as a multivariate *P* <0.05. The odds ratios and their 95% confidence intervals (CIs) from the multivariate logistic regression analysis were used as estimates of relative risk. Kaplan-Meier survival curves for components of MACE and overall MACE rate were constructed and compared between groups with the Log-Rank test. Multivariate Cox proportional hazard analysis was used to determine the independent predictors of TVR and overall MACE after adjustment for baseline and angiographic variables with unequal distribution. A p-value <0.05 was considered significant for all analyses. Statistical analysis was done using SPSS 11.5 or SAS 9.3.

## Results

### Patient characteristics

The demographic data of both groups of patients are listed in Table 1. Patients in the lower-TG group were older (67 vs. 56 years, p < 0.01) and had lower body mass index, worse estimated glomerular filtration rate, higher high-density lipoprotein (HDL) as well as lower total cholesterol levels than those in the higher-TG group.

**Table 1:** **Baseline characteristics of patients in the lower**-**TG** (≦**150 mg**/**dl**) **and higher**-**TG** (>**150 mg**/**dl**) **groups**

	Lower-TG group	Higher-TG group	P
**N**	163	84	
**Age,** **yrs**	67.1 ± 13.2	56.1 ± 11.3	<0.001
**Female** **(%)**	22 (13.6)	19 (22.6)	0.100
**Hypertension** **(%)**	97 (59.5)	61 (72.6)	0.058
**DM** **(%)**	46 (28.2)	34 (40.5)	0.071
**Smoking** **(%)**	99 (60.7)	56 (66.7)	0.439
**BMI,** **kg/m** ^**2**^	24.1 ± 3.5	26.3 ± 3.2	<0.001
**Previous CAD** **(%)**	22 (13.5)	5 (6.0)	0.113
**Previous stroke** **(%)**	15 (9.2)	5 (6.0)	0.522
**Carotid stenosis** **(%)**	4 (2.5)	0 (0)	0.303
**PAD** **(%)**	3 (1.8)	2 (2.4)	1.000
**eGFR,** **ml/min**	46.7 ± 18.5	57.6 ± 20.5	<0.001
**Previous hypolipid drugs**			
**Statin**	21 (12.9)	12 (14.3)	0.913
**Fibrate**	1 (0.6)	3 (3.6)	0.115
**Admission lipid profile**			
**HDL,** **mg/dl**	45.4 ± 10.8	37.4 ± 8	<0.001
**LDL,** **mg/dl**	100.9 ± 31.6	106.6 ± 41.4	0.231
**TC/HDL**	3.65 ± 0.87	5.02 ± 1.36	<0.001
**LDL/HDL**	2.32 ± 0.81	2.93 ± 1.28	<0.001
**TG,** **mg/dl** **(range)**	83.6 ± 34.3 (13–149)	258.6 ± 136.8 (152–805)	<0.001
**Cardiogenic shock** **(%)**	29 (17.8)	7 (8.3)	0.071

*BMI*, body mass index; *CAD*, coronary artery disease; *DM*, diabetes mellitus; *eGFR*, estimated glomerular filtration rate; *HDL*, high-density lipoprotein; *LDL*, low-density protein; *PAD*, peripheral arterial disease; *TC*, total cholesterol; *TG*, triglyceride.

### ECG, coronary angiographic findings, pPCI results and in-hospital outcomes

The territory of myocardial infarction determined by ECG was mostly located in the anterior wall in both groups (Table 2). The severity of overall coronary artery disease, the culprit lesion vessel, the ECG-to-balloon time and the therapeutic modalities of pPCI in terms of balloon angioplasty, thrombectomy, and endovascular stenting were similar between the two groups (p = NS in all). Success of PCI (post-procedural TIMI-blood flow to ≧grade 2) was accomplished in most of the patients in both groups (p = NS). Though post-procedural left ventricular ejection fraction estimated with either ventriculography or echocardiography was more depressed in the low-TG group (44.8% vs. 46.9%, p = 0.031), myocardial infarct size estimated by peak creatinine kinase (CK) levels was comparable between the two groups. Occurrence of new cardiogenic shock, respiratory failure, atrial fibrillation and ventricular arrhythmia, as well as requirement of emergency coronary bypass surgery was also statistically equivalent in the two groups (p = NS in all). Though in-hospital mortality happened in 6 patients in the lower TG (5 from ventricular arrhythmia and 1 from refractory pumping failure) but 0 in the higher TG group, the difference was not statistically significant (p = 0.098). Further univariate followed by multivariate regression analysis identified peak CK and CAD number >1 as positive, whereas serum TG level as negative predictors of in-hospital death for all of these patients (Table 3).

**Table 2:** **ECG location of STEMI**, **findings of coronary angiograms and results of primary percutaneous coronary interventions in all patients**

	Lower-TG group	High-TG group	P
**N**	**163**	**84**	
**ECG infarct area**			
**Anterior** **(%)**	**87** **(53.4)**	**51** **(** **60.7** **)**	**0.334**
**Inferior** **(%)**	**72** **(** **44.2** **)**	**33** **(** **39.3** **)**	**0.548**
**True posterior** **(%)**	**2** **(** **1.2** **)**	**2** **(** **2.4** **)**	**0.607**
**High lateral** **(%)**	**8** **(** **4.9** **)**	**4** **(** **4.8** **)**	**1.000**
**Severity of CAD**			
**Number of diseased vessels**	**1.8** **±** **0.8**	**1.8** **±** **0.8**	**0.906**
**SVD** **(%)**	**76** **(** **46.6** **)**	**37** **(** **44.0** **)**	**0.802**
**DVD** **(%)**	**50** **(** **30.7** **)**	**30** **(** **35.7** **)**	**0.501**
**TVD** **(%)**	**37** **(** **22.7** **)**	**17** **(** **20.2** **)**	**0.779**
**LM involvement** **(%)**	**7** **(** **4.3** **)**	**1** **(** **1.2** **)**	**0.271**
**Treated** **(** **culprit** **)** **vessel**			
**LM** **(%)**	**2** **(** **1.2** **)**	**1** **(** **1.2** **)**	**1.000**
**LAD** **(%)**	**86** **(** **52.8** **)**	**51** **(** **60.7** **)**	**0.291**
**LCx** **(%)**	**18** **(** **11** **)**	**6** **(** **7.1** **)**	**0.451**
**RCA** **(%)**	**57** **(** **35** **)**	**28** **(** **33.3** **)**	**0.908**
**Ramus medianus** **(%)**	**1** **(** **0.6** **)**	**0** **(** **0** **)**	**1.000**
**Door to balloon time,** **min**	**90.5** **±** **42.1**	**87.7** **±** **29**	**0.587**
**Interventions**			
**Balloon angioplasty** **(%)**	**161** **(** **98.8** **)**	**81** **(** **96.4** **)**	**0.341**
**Thrombectomy**			
**Aspiration** **(%)**	**103** **(** **63.2** **)**	**55** **(** **65.5** **)**	**0.83**
**Rheolytic** **(%)**	**3** **(** **1.8** **)**	**2** **(** **2.4** **)**	**1.000**
**Stenting**			
**Nil** **(%)**	**15** **(** **9.2** **)**	**10** **(** **11.9** **)**	**0.657**
**DES** **(%)**	**37** **(** **22.7** **)**	**21** **(** **25.0** **)**	**0.806**
**BMS** **(%)**	**108** **(** **66.3** **)**	**52** **(** **61.9** **)**	**0.591**
**DES** **+** **BMS** **(%)**	**3** **(** **1.8** **)**	**1** **(** **1.2** **)**	**1.000**
**Final TIMI flow**			
**Grade 3** **(%)**	**153** **(** **93.9** **)**	**82** **(** **97.6** **)**	**0.231**
**Grade 2** **(%)**	**10** **(** **6.1** **)**	**2** **(** **2.4** **)**	**0.231**
**Grade 1/0** **(%)**	**0** **(** **0** **)**	**0** **(** **0** **)**	**0**
**In-hospital outcome**			
**LVEF, %**	**43.8** **±** **10.9**	**46.9** **±** **9.5**	**0.031**
**Cardiac markers**			
**Peak CK,** **mg/dl**	**2683.3** **±** **2451.8**	**2735.1** **±** **2676.8**	**0.897**
**Complications**			
**New cardiogenic shock** **(%)**	**21** **(** **12.9** **)**	**6** **(** **7.1** **)**	**0.248**
**Respiratory failure** **(%)**	**30** **(** **18.4** **)**	**8** **(** **9.5** **)**	**0.100**
**Ventricular arrhythmia** **(%)**	**25** **(** **15.3** **)**	**8** **(** **9.5** **)**	**0.282**
**New atrial fibrillation** **(%)**	**16** **(** **9.8** **)**	**3** **(** **3.6** **)**	**0.142**
**Emergency CABG** **(%)**	**0** **(** **0** **)**	**2** **(** **2.4** **)**	**0.115**
In hospital Death* **(%)**	**6** **(** **3.7** **)**	**0** **(** **0** **)**	**0.098**

*All were related with refractory heart failure, with 5 of them experiencing ventricular arrhythmia before death. *BMS*, bare-metal stent; *CABG*, coronary artery bypass grafting surgery; *CAD*, coronary artery disease; *CK*, creatinine kinase; *DES*, drug-eluting stent; *DVD*, double vessel disease; *LAD*, left anterior descending artery; *LCx*, left circumflex artery; *LM*, left main coronary artery; *RCA*, right coronary artery; *SVD*, single vessel disease; *TIMI*, thrombolysis in myocardial infarction; *TVD*, triple vessel disease.

**Table 3:** **Independent predictors of in**-**hospital mortality in patients with STEMI undergoing pPCI**

Variable	Hazard ratio	95% C.I.	P*	P ^†^
**Variables in the model**				
CAD number	4.620	1.006-21.223	0.038	0.049
1 (reference)	1			
>1	4.620	1.006-21.223	0.038	0.049
Peak CK	1.001	1.000-1.001	0.001	0.003
TG	0.963	0.931-0.995	0.049	0.023
**Variables not in the model**				
Age			0.19	
Male			0.794	
Hypertension			0.339	
DM			0.419	
Cholesterol			0.047	NS
ECG to balloon time			0.753	
LM involvement			0.102	NS
Cardiogenic shock			0.004	NS
Anterior infarction			0.770	
Culprit LAD			0.286	
Culprit LCx			0.844	
Culprit RCA			0.424	
Culprit LM			0.015	NS
TIMI grade			<0.001	NS
Thrombectomy				
Aspiration			0.339	
Rheolytic			0.040	NS

*From univariate regression analysis, ^†^from multivariate regression analysis. *CAD*, coronary artery disease; *CK*, creatinine kinase; *DM*, diabetes mellitus; *LAD*, left anterior descending artery; *LCx*, left circumflex artery; *LM*, left main coronary artery; *RCA*, right coronary artery; *TIMI*, thrombolysis in myocardial infarction; *TG*, triglyceride.

### Long-term outcomes

The medications prescribed at discharge were similar in the two groups of patients surviving the STEMI episodes, except that fibrates were given more often in higher-TG patients with the intention to lower serum TG level (Table 4). Kaplan-Meier survival test showed that during a mean follow-up period of 1.23 years for lower-TG and 1.4 years for higher-TG patients (p = 0.126), lower-TG patients had significantly more incidences of TVR (21.7% vs. 9.5%, Log-Rank p = 0.0111) and in turn overall MACE (26.1% vs. 11.9%, Log-Rank p = 0.0137) compared to higher-TG patients, yet the rates of de novo lesions, non-fatal MI, cardiac deaths and all-cause mortality were comparable between groups (Table 4). Multivariate Cox proportional hazard model confirmed that, besides the number of diseased coronary arteries as a positive predictor, serum TG level inversely correlated with TVR (hazard ratio 0.993, 95% CI 0.988-0.998, p = 0.007) and overall MACE (hazard ratio 0.994, 95% CI 0.990-0.999, p = 0.016) in all of our study patients (Table 5).

**Table 4:** **Take**-**home medications and late clinical outcomes**

	Lower-TG group	Higher-TG group	P
**N**	157	84	
**Take-home medications**			
**Aspirin** **(%)**	153 (97.5)	84 (100.0)	0.301
**Clopidogrel** **(%)**	154 (98.1)	83 (98.8)	1.000
*RAAS inhibitor **(%)**	145 (92.4)	77 (91.7)	1.000
**Beta Blocker** **(%)**	77 (47.2)	47 (56.0)	0.375
**Statin** **(%)**	91 (58)	54 (64.3)	0.414
**Fibrate** **(%)**	2 (1.3)	11 (13.1)	<0.001
**Follow-up years** **(mean)**	1.23 (0.22-3.78)	1.40 (0.44-3.73)	0.126
**MACE**			
**Non-fatal MI** **(%)**	17 (10.8)	3 (3.6)	0.0731^†^
**TVR** **(%)**	34 (21.7)	8 (9.5)	0.0111^†^
**De novo lesion** **(%)**	9 (5.7)	4(4.8)	0.404^†^
**Cardiac Death** **(%)**	7 (4.5)	0 (0)	0.1688^†^
**All-Cause Mortality** **(%)**	13 (8.3)	1 (1.2)	0.1392^†^
**Overall** **(%)**	41 (26.1)	10 (11.9)	0.0137^†^

*Including angiotensin-converting-enzyme inhibitors and angiotensin-II receptor blockers. ^†^derived from Log-Rank test. *MACE*, major adverse cardiovascular events; *MI*, myocardial infarction; *RAAS*, rennin-angiotensin-aldosterone system; *TVR*, target vessel revascularization.

**Table 5:** **Independent predictors of TVR and overall MACE by multivariate Cox regression analysis in all patients**

Variable	Hazard ratio	95% C.I.	P
**TVR**			
TG*	0.993	0.988-0.998	0.007
CAD number*			
**1** **(** **reference** **)**	1		
**>1**	2.717	1.655-4.458	<0.001
**Overall MACE**			
TG*	0.994	0.990-0.999	0.016
CAD number*			
**1** **(** **reference** **)**	1		
**>1**	2.706	1.730-4.234	<0.001

*Adjusted for age, gender, smoking, diabetes mellitus, left ventricular ejection fraction, peak creatinine kinase level, balloon angioplasty only, bare-metal stent, drug-eluting stent, low-density lipoprotein level, all medications and high-density lipoprotein level. *CAD*, coronary artery disease; *MACE*, major adverse cardiovascular event; *TG*, triglyceride; *TVR*, target vessel revascularization.

## Discussion

Patients with coronary artery disease presenting as STEMI might develop early fatal/non-fatal complications and late MACE in terms of target lesion revascularization, non-fatal myocardial infarction and cardiac death despite being successfully treated by pPCI. This study, which investigated the possible relations between serum TG and in-hospital as well as late outcomes of such patients, indicated that serum TG level correlates negatively with in-hospital mortality: TG level above 150 mg/dl predicts fewer incidences of late TVR and in turn overall MACE. These findings illustrate the paradoxically inverse relation between serum TG level and clinical outcome of patients with STEMI treated with pPCI, and may justify exclusion of specific TG-lowering therapy from the standard therapeutic regimen in management of such patients in the long term.

### Role of TG in in-hospital outcome of STEMI

The role of serum TG in the pathogenesis of CAD is not as clear as that of serum LDL. A number of clinical studies reported a positive association between TG level and severity of CAD [7, 15–17], whereas others demonstrated conflicting results [18–20]. Investigations involving histological research found that triglycerides played a constitutive role in atherosclerotic plaques yet were rarely identified in active coronary artery lesions as a culprit component [21]. These findings suggest that triglycerides might be merely an innocent bystander rather than a direct atherogenic mediator [22–24], and this concept may offer some explanation for our findings that patients with higher TG levels are not at risk of poorer in-hospital outcome after STEMI. In fact, our results demonstrate that there exists a paradoxical trend toward more in-hospital deaths in patients with lower TG levels, though the between-group difference did not reach statistical significance possibly due to limited patient number and confounding by coexistent variables. The role that serum TG plays as an independent negative predictor for in-hospital deaths, finally shown by multivariate regression analysis in this study, has similarly been reported in patients with acute stroke [11–13] and acute coronary syndrome [25], but is not easy to understand compared to the roles played by CK level and CAD number as positive predictors, since these two variables represent surrogates of infarct size and ischemic myocardial territory thus are rationally related with in-hospital mortality. One possible explanation for the inverse relation between TG level and in-hospital mortality comes from the findings that incidences of new atrial arrhythmia, ventricular arrhythmia, respiratory failure and new cardiogenic shock all tended to be higher in the lower TG group, along with the exclusive occurrence of lethal ventricular arrhythmia and refractory heart failure in 6 (3.7%) of lower TG patients but not in any of higher TG group. These results implicate the potential role of serum TG in stabilizing the infarcted, stunned or reperfused myocardium after an acute STEMI episode. Another possibility may be based on the concept that serum TG level is an indicator of nutritional status. Lower TG levels denote poorer nutritional condition which could halt myocardial and whole body recovery from STEMI, in turn subjecting patients to in-hospital deaths. This hypothesis gains support from some epidemiologic studies describing low body mass index and serum cholesterol level as markers of poor health status in older subjects [26–28], and from clinical investigations reporting worse outcome in patients with advanced heart failure or various cardiovascular diseases and low serum cholesterol [29, 30] or TG concentrations [11–13]. Further studies are needed to determine the exact pathophysiological relations between serum TG level and in-hospital outcome in patients with STEMI who receive pPCI treatment.

### Role of TG in late outcome of STEMI

Previous studies have demonstrated that even after primary PCI a considerable proportion of patients with STEMI still succumb to subsequent MACE due to left ventricular remodeling and coronary restenosis [31]. The distinct pathogenesis of restenosis caused by smooth muscle cell proliferation rather than lipid-related neo-atherosclerosis [32] explains the poor preventive efficacy of lipid-lowering medications, particularly statins, on inhibition of neointimal formation in this entity of lesions [33–35]. Nonetheless, whether higher TG-rich lipoprotein is [36, 37] or is not [34] related with the restenotic phenomenon remains debatable. The current study demonstrated that risk of de novo atherosclerosis was similar between groups with higher or lower TG levels but overall MACE rates, contributed mostly from incidence of TVR, were significantly lower in high-TG patients. These results suggest a potential action of serum TG against neointimal proliferation at the STEMI-related lesion sites ever treated by angioplasty or stenting. This phenomenon has ever been reported in an in vitro study describing the distinct effects of various TG particles on either stimulation or suppression of the proliferative activity of vascular smooth muscle cells [38]. Our findings further indicate that though hypertriglyceridemia is regarded as an independent risk factor for atherosclerosis [39] when coexistent with low HDL- and elevated LDL-cholesterol and needs to be treated after the LDL goal is reached [25], application of such a therapeutic strategy to patients with STEMI post-pPCI is not warranted. On the contrary, the paradoxically inverse relation between hypertriglyceridemia and better late outcome in patients with STEMI treated with pPCI may justify exclusion of specific TG-lowering therapy from the standard therapy in long-term management of such patients. Whether there exists a “J-curve relationship” between serum TG level and the outcome of patients with STEMI treated with pPCI awaits further clarification.

## Conclusion

On-admission serum TG levels inversely correlate with in-hospital deaths of patients with STEMI treated with pPCI, and those with TG levels below 150 mg/dl have a worse long-term outcome in terms of TVR and overall MACE than those with higher levels. Thus, serum TG can be regarded as a mediator that protects such patients from short and late cardiovascular events rather than a hazard factor, and hence does not warrant aggressive pharmacological treatment to lower it to the so-called normal level.

## Abbreviations

CAD: Coronary artery disease
CK: Creatinine kinase
HDL: High-density lipoprotein
LDL: Low-density lipoprotein
MACE: Major adverse cardiovascular event
pPCI: Primary percutaneous coronary intervention
STEMI: ST-segment elevation myocardial infarction
TIMI: Thrombolysis in myocardial infarction
TG: Triglyceride
TVR: Target vessel revascularization.
